# A Nonribosomal Peptide Synthase Gene Driving Virulence in Mycobacterium tuberculosis

**DOI:** 10.1128/mSphere.00352-18

**Published:** 2018-10-31

**Authors:** Kiranmai Bhatt, Henrique Machado, Nuno S. Osório, Jeremy Sousa, Filipa Cardoso, Carlos Magalhães, Bing Chen, Mei Chen, John Kim, Albel Singh, Catarina M. Ferreira, António G. Castro, Egidio Torrado, William R. Jacobs, Apoorva Bhatt, Margarida Saraiva

**Affiliations:** a Heart of England NHS Trust, Birmingham, United Kingdom; b ICVS, University of Minho, Braga, Portugal; c ICVS/3B’s–PT Government Associate Laboratory, Braga, Portugal; d i3S–Instituto de Investigação e Inovação em Saúde, Porto, Portugal; e IBMC–Instituto de Biologia Molecular e Celular, Universidade do Porto, Porto, Portugal; f Department of Microbiology and Immunology, Albert Einstein College of Medicine, New York, New York, USA; g School of Biosciences and Institute of Microbiology and Infection, University of Birmingham, Birmingham, United Kingdom; h Howard Hughes Medical Institute, Albert Einstein College of Medicine, New York, New York, USA; Washington University in St. Louis School of Medicine

**Keywords:** immune deficiency, pathogenesis, tuberculosis, virulence factors

## Abstract

Nonribosomal peptide synthases produce short peptides in a manner that is distinct from classical mRNA-dependent ribosome-mediated translation. The Mycobacterium tuberculosis genome harbors a nonribosomal peptide synthase gene, *nrp*, which is part of a gene cluster proposed to be involved in the biosynthesis of isonitrile lipopeptides. Orthologous clusters are found in other slow-growing pathogenic mycobacteria and actinomycetes. To probe the role of the *nrp* gene in infection, we generated an *nrp* deletion mutant in M. tuberculosis H37Rv and tested its virulence in immunocompetent (C57BL/6) mice. The *nrp* mutant strain displayed lower initial growth rates in the lungs and a defective dissemination to the spleens of infected mice. Mice infected with the mutant strain also survived for twice as long as those infected with wild-type M. tuberculosis and, remarkably, showed subdued pathology, despite similar bacterial loads at later stages of infection. The differences in the course of infection between wild-type and *nrp* mutant strains were accompanied by distinct dynamics of the immune response. Most strikingly, the *nrp* mutant was highly attenuated in immunodeficient (SCID-, recombination activating 2 [RAG2]-, and gamma interferon [IFN-γ]-deficient) mice, suggesting that macrophages control the *nrp* mutant more efficiently than they control the wild-type strain. However, in the presence of IFN-γ, both strains were equally controlled. We propose that the *nrp* gene and its associated cluster are drivers of virulence during the early stages of infection.

**IMPORTANCE** Over 10 million people developed tuberculosis (TB) in 2016, and over 1.8 million individuals succumbed to the disease. These numbers make TB the ninth leading cause of death worldwide and the leading cause from a single infectious agent. Therefore, finding novel therapeutic targets in Mycobacterium tuberculosis, the pathogen that causes most cases of human TB, is critical. In this study, we reveal a novel virulence factor in M. tuberculosis, the *nrp* gene. The lack of *nrp* highly attenuates the course of M. tuberculosis infection in the mouse model, which is particularly relevant in immune-deficient hosts. This is very relevant as TB is particularly incident in immune-suppressed individuals, such as HIV patients.

## Abstract (precis)

Over 10 million people developed tuberculosis (TB) in 2016, and over 1.8 million individuals succumbed to the disease. These numbers make TB the ninth leading cause of death worldwide and the leading cause from a single infectious agent. Therefore, finding novel therapeutic targets in Mycobacterium tuberculosis, the pathogen that causes most cases of human TB, is critical. In this study, we reveal a novel virulence factor in M. tuberculosis, the *nrp* gene. The lack of *nrp* highly attenuates the course of M. tuberculosis infection in the mouse model, which is particularly relevant in immune-deficient hosts. This is very relevant as TB is particularly incident in immune-suppressed individuals, such as HIV patients.

## INTRODUCTION

Tuberculosis (TB) remains a leading cause of death by an infectious agent. Indeed, TB, caused by Mycobacterium tuberculosis, kills over 1.8 million people, afflicts over 10 million new individuals per year, and is estimated to exist in a latent form in one-fourth of the world’s population (1). To reach the milestone of less than one TB case per million individuals, set by the World Health Organization (WHO) for 2050, numerous roadblocks will have to be overcome, notably, the development of more efficient vaccines and therapies. Success on these fronts requires a greater understanding of the mechanisms underlying the success of M. tuberculosis as a pathogen. In this regard, a variety of genetic strategies have been employed to unlock novel virulence factors in M. tuberculosis and hence druggable targets (2–4).

Nonribosomal peptide synthases (NRPSs) are large multifunctional proteins involved in the biosynthesis of short peptides in a manner that is distinct from the ribosome-mediated translation of mRNA (5). The genes encoding NRPSs are predominantly found in bacteria and fungi that produce secondary metabolites (6). The short peptides produced by NRPSs usually form part of a larger more complex molecule. The genomes of mycobacteria, including the TB-causing M. tuberculosis, contain genes encoding NRPSs. While NRPSs in environmental bacteria are often associated with antibiotic and other secondary metabolite production (6), mycobacterial NRPSs are mainly involved in the production of components of complex cell wall lipids and siderophores (7, 8). *Rv0101* (*nrp*) is an M. tuberculosis H37Rv gene annotated as a NRPS (9). The gene encodes a putative bimodular NRPS containing seven catalytic domains (10, 11), including an adenylation domain with predicted substrate specificity for lysine and phenylalanine. *nrp* is located in a cluster of genes with potentially linked functions: *Rv0097* encodes a putative oxidase, *Rv0098* encodes a long-chain fatty acyl-coenzyme A (fatty acyl-CoA) thioesterase (12, 13), and FadD10 (*Rv0099*) is a fatty acyl-AMP ligase that catalyzes the transfer of fatty acyl chains to an acyl carrier protein encoded by *Rv0100* (10). The knowledge about the precise function of *nrp* and its associated cluster is based solely on the structural characterization and *in vitro* enzyme assays with heterologously expressed and purified M. tuberculosis and Mycobacterium marinum proteins and suggests a role in the production of a novel lipopeptide with isonitrile functionality (10, 14).

Importantly, several *in vitro* and *in vivo* infection studies have highlighted a potential role for *nrp* in the pathogenesis of TB. Specifically, *nrp* was reported as a nonessential gene for *in vitro* growth by transposon mutagenesis (15) but was otherwise required for growth in the spleens of C57BL/6J mice (16). Additionally, the gene cluster spanning *Rv0096-Rv0101* was shown to be critical for survival in mice (17). Furthermore, Nrp was reported to be the most abundant M. tuberculosis protein in the lungs of infected guinea pigs by day 30 postinfection, while being undetected at 90 days postinfection (18). Overall, these reports indicate that the cryptic metabolite produced by the *nrp* cluster is likely to play a role in virulence, which calls for a deeper investigation of *nrp* in the context of M. tuberculosis biology and infection.

In this study, we sought to query the role of the *nrp* gene in the pathogenesis of TB in the mouse model of infection. Our strategy consisted of the generation of M. tuberculosis mutant strains lacking the *nrp* gene. We herein reveal an important role for *nrp* in the virulence of M. tuberculosis, likely mediating the initial interaction of the bacteria with the host macrophages. We show that *nrp*-deficient M. tuberculosis grows at a lower rate in immunocompetent and immunodeficient mice, causing less lung pathology and associated with significantly increased survival rates.

## RESULTS

### *nrp* cluster in mycobacteria.

**FIG 1:**
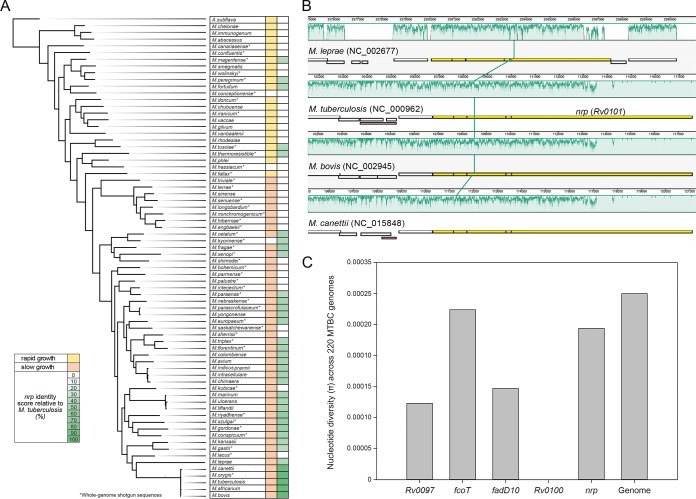
The *nrp* cluster is highly conserved among the MTBC and slow-growing species of the genus *Mycobacterium*. (A) Sequence identity scores of the *nrp* cluster were obtained by querying the M. tuberculosis H37Rv sequences *Rv0097-Rv0101* against a local database of 72 genomes of species representative of the phylogeny of the genus *Mycobacterium* (19). The genomics sequences include complete genomes and whole-genome shotgun sequences. The highest percentage (dark green) corresponds to the longest and most elevated sequence identities. (B) Synteny of the genome sections harboring the *nrp* cluster in M. tuberculosis, M. bovis, M. canettii, and M. leprae. The green shading indicate homologous DNA regions shared by the four chromosomes without major sequence rearrangements. The genes of the *nrp* cluster are highlighted in yellow. (C) Nucleotide diversity (π) level of the M. tuberculosis genes *Rv0097*, *fcoT*, *fad10*, *Rv0100*, and *nrp* and the complete genome across a set of 220 genomes, including strains from lineage 1 to 7 of the MTBC.

The M. tuberculosis *nrp* gene (*Rv0101*) encodes a bimodular NRPS with domains predicted to activate Lys and Phe (10, 11, 14). The gene is present in a cluster of 5 genes including *nrp* plus *Rv0097* to *Rv0100*. A sequence comparison analysis of the *nrp* cluster among 72 genomes from species representative of the phylogeny of the genus *Mycobacterium* (19) revealed that this cluster is absent in most rapid-growing nontuberculous mycobacteria (NTM), commonly found in the environment and considered nonpathogenic (Fig. 1A). The NTM exceptions harboring genes of the *nrp* cluster with more than 40% identity score to the genes of M. tuberculosis were Mycobacterium peregrinum, Mycobacterium fortuitum, Mycobacterium mageritense, Mycobacterium tusciae, and Mycobacterium thermoresistible. Most slow-growing mycobacteria (31 of 48 [64.6%]) harbor orthologs to the M. tuberculosis *Rv0097-Rv0101* genes with identity scores above 45% (Fig. 1A). This includes other pathogenic species, such as Mycobacterium leprae (50.6% identity) and Mycobacterium ulcerans (55.2% identify) (Fig. 1A). A synteny analysis showed that the *nrp* cluster is located in a homologous genomic DNA region shared by the chromosomes of the species of the Mycobacterium tuberculosis complex (MTBC) and M. leprae, without major sequence rearrangements. The exception is the considerably smaller size of the *nrp* gene in M. leprae compared to the *nrp* genes found in species from the MTBC (4,206 bp in M. leprae NC_002677 versus 7,539 bp in M. tuberculosis NC_000962) (Fig. 1B). As a result of this difference, the structural prediction is that the *nrp* gene found in MTBC strains encodes a bimodular NRPS, whereas the homologues from other mycobacteria, such as M. leprae, encode a single NRPS module. A detailed analysis of the level of nucleotide diversity of these genes within the MTBC was obtained by the analysis of 220 M. tuberculosis genomes from 7 MTBC lineages (20) and showed that the level of nucleotide diversity of these genes is below the genome-wide level (Fig. 1C). Thus, the comparative genome analysis suggests that the *nrp* cluster is highly conserved among slow-growing members of the *Mycobacterium* genus, highlighting the larger size of the *nrp* gene as the most characteristic MTBC-associated feature.

### Loss of the *nrp* gene results in attenuation of M. tuberculosis H37RV in mice.

To investigate the role of *nrp* in the virulence of slow-growing mycobacteria, we first generated a null mutant of M. tuberculosis H37Rv *nrp* by using specialized transduction to replace *nrp* with a hygromycin resistance cassette. Subsequently, we generated a complemented strain (*nrp*-comp) by introducing an integrative shuttle cosmid vector (containing an M. tuberculosis H37Rv cosmid fragment spanning *Rv0096* to *Rv0109*) into the Δ*nrp* mutant (see Fig. S1A in the supplemental material). To control for possible polar effects of the Δ*nrp* deletion on the adjacent gene *Rv0102*, the expression of this gene was monitored in either of the recombinant M. tuberculosis strains. We found that the expression patterns of *Rv0102* were similar in mutant and complemented bacteria (Fig. S1B). Furthermore, the axenic growth of the two engineered M. tuberculosis H37Rv strains was identical and similar to that of the wild-type (WT) H37Rv (Fig. S1C), showing no major growth defects imposed by the absence of *nrp*. Additionally, the Δ*nrp* strain retained its acid-fast properties (Fig. S1D).

10.1128/mSphere.00352-18.1 FIG S1 (A) Generation of Δ*nrp* and *nrp*-comp strains. Map of pYUB2420 indicating *nrp* cluster genes included in cloned cosmid fragment (top). Generation of M. tuberculosis H37Rv Δ*nrp* and Δ*nrp*-comp strains (bottom). Maps of the M. tuberculosis *nrp* region in wild-type (H37Rv), Δ*nrp*, and *nrp*-comp strains: [α ^32^P]dCTP-labeled probes were derived from ∼1-kb upstream and downstream flanking sequences that were used to construct the knockout plasmids and are indicated by thick lines with circles at each end (bottom left). The fragments expected to hybridize with the probes in a Southern blot are indicated by red lines with sizes indicated. *res*, γδ-resolvase site; *hyg*, hygromycin resistance gene. Southern blot of BamHI digested genomic DNA from H37Rv Δ*nrp* and *nrp*-comp strains (bottom right). (B) Δ*nrp* and *nrp*-comp strains were grown to mid-log phase, and RNA was extracted by acid phenol and chloroform extraction. The purified RNA was treated with DNase and converted to cDNA, which was used as a template for quantitative PCR. PCR products were run on a 1.5% agarose gel to detect the expression of *Rv0102*. *sigA* was used as a housekeeping gene. Relative values between the two genes were measured by densitometry. (C) Axenic growth (in liquid 7H9 medium) of H37Rv (WT), Δ*nrp*, and *nrp*-comp strains. Biomass was measured over time by the optical density at 570 nm over a 12-day period. (D) Deletion of *nrp* does not affect acid-fast properties of M. tuberculosis, as determined by Ziehl-Neelsen staining of lungs infected for 30 days with Δ*nrp* or *nrp*-comp strains. Download FIG S1, TIF file, 1.0 MB. Copyright © 2018 Bhatt et al. 2018 Bhatt et al. This content is distributed under the terms of the Creative Commons Attribution 4.0 International license.

**FIG 2:**
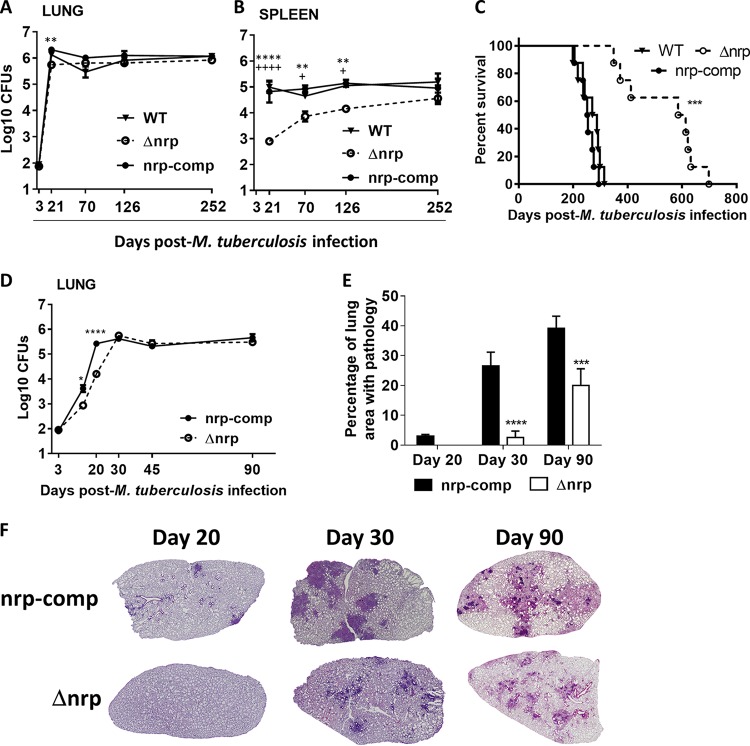
Loss of the *nrp* gene results in attenuation of M. tuberculosis H37Rv in mice. C57BL/6 mice were infected by aerosol exposure to H37Rv (WT), Δ*nrp*, or *nrp*-comp strains. At the indicated time points, the lungs (A and D) and spleens (B) of infected mice were collected and the bacterial burden determined by CFU enumeration. (C) The weights of the animals were monitored to determine survival curves. On days 20, 30, and 90 postinfection, lung pathology (E and F) was determined by hematoxylin and eosin (H&E) staining and morphometric analysis of the right upper lobes of infected lungs. Data are shown as the means ± standard errors of the means (SEMs) from 5 independent animals in at least 2 independent experiments. The pictures in panel F are of one animal representative of the experimental group; ×40 magnification. Statistical analysis was performed with a two-way ANOVA using Sidak’s test for multiple comparisons (A, B, D, and E) or with log-rank (Mantel-Cox) test for the Kaplan-Meier curves (C). * and + refer to statistical differences between Δ*nrp* and *nrp*-comp strains or Δ*nrp* and WT strains, respectively. * or +, *P* < 0.05; **, *P* < 0.01; ***, *P* < 0.001; **** or ++++, *P* < 0.0001.

To assess the role of *nrp* in the pathogenesis of M. tuberculosis in the murine model of infection, immunocompetent (C57BL/6) mice were infected with aerosols of WT, Δ*nrp*, or *nrp*-comp strains of M. tuberculosis H37Rv. Bacterial burdens in the lungs and spleens of infected mice were analyzed at different time points postinfection (Fig. 2A and B). At 3 weeks postinfection, fewer colonies of the Δ*nrp* strain were found in infected lungs than of the WT or *nrp*-comp strains (Fig. 2A). However, the lung bacterial loads of all three strains were similar at later time points (Fig. 2A), indicating that the Δ*nrp* strain eventually grew to a level comparable to that of the WT strain in the long term. The differences in bacterial loads in the spleen were stark between the strains, with considerably fewer bacteria cultured from the spleens of mice infected with the Δ*nrp* strain (Fig. 2B). Furthermore, we observed a significant difference in the survival of mice infected with the different strains. While all mice in groups infected with WT or *nrp*-comp strains died prior to 325 days postinfection, those infected with the Δ*nrp* strain survived for up to 700 days postinfection (Fig. 2C). Given that we were able to complement the growth phenotypes in C57BL/6 (Fig. 2A to C), we chose to focus our comparison of the Δ*nrp* and *nrp*-comp strains for all further studies described below in an effort to limit the number of mice used in our experiments.

To further explore the growth kinetics of the Δ*nrp* strain in the early stages of infection, we repeated the infection of C57BL/6 mice to monitor CFU levels in lungs from day 14 to up to 90 days postinfection. The difference in CFU between Δ*nrp*-strain- and *nrp*-comp-strain-infected mice was readily visible on day 14 postinfection, corresponding to a 48-fold lower bacterial burden (1.683 log_10_ average difference) in the case of mice infected with the Δ*nrp* strain on day 20. From day 30 postinfection and up to day 90, the Δ*nrp* strain reached lung bacillary counts comparable to those of the *nrp*-comp strain (Fig. 2D), as observed before (Fig. 2A).

### Δ*nrp*-strain-infected mice develop less lung pathology.

In addition to the differences observed in the progression of bacterial burdens, a distinct phenotype was also observed in lung histopathological features during infection with the Δ*nrp* strain versus the *nrp*-comp strain (Fig. 2E and F). Specifically, by day 20 postinfection, no discernible lesions were observed in the lungs of Δ*nrp*-strain-infected animals, whereas multiple small inflammatory infiltrates were already present in the lungs of animals infected with the *nrp*-comp strain (Fig. 2E and F). This was likely due to the differences in bacterial burdens observed at this time point (Fig. 2D). By day 30 postinfection, the lesions in the lungs of mice infected with the *nrp*-comp strain were larger than those in mice infected with the Δ*nrp* strain (Fig. 2E and F), even though both presented the same bacterial burden (Fig. 2D). Remarkably, the histological differences were maintained throughout the chronic stage of infection, as on day 90 postinfection, the animals infected with the *nrp*-comp strain still presented a greater degree of pathology than those infected with the Δ*nrp* strain (Fig. 2E and F), despite the fact that the bacterial burdens had been similar in both cases for 60 days (Fig. 2D). This subdued pathology may explain the longer survival of mice infected with the Δ*nrp* strain (Fig. 2C).

### Kinetics of the immune response is altered in Δ*nrp*-strain-infected mice.

**FIG 3:**
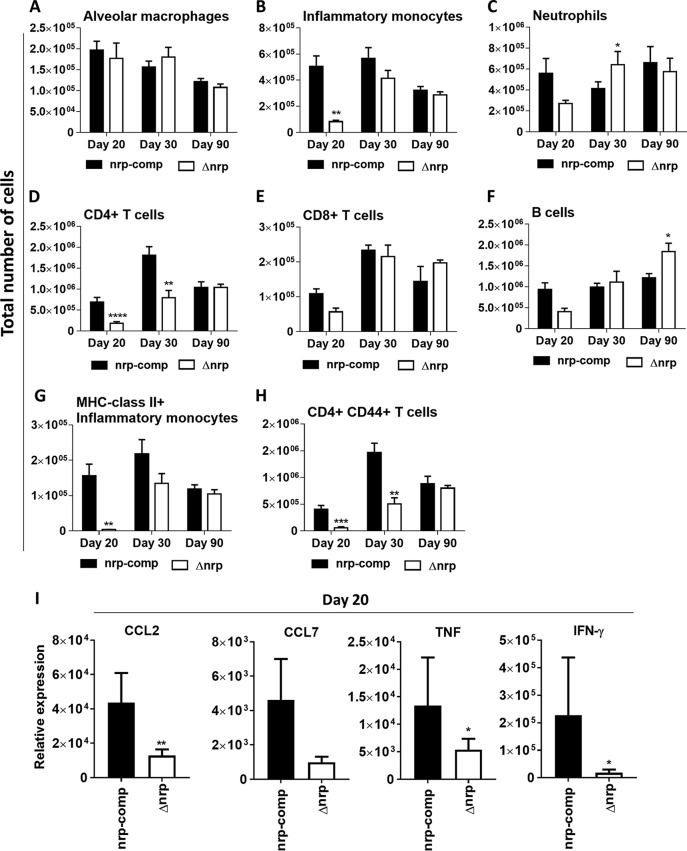
Lack of *nrp* alters the dynamics of the lung immune response to infection. (A to H) At the indicated time points postinfection, the lungs of C57BL/6 mice infected via aerosol exposure to Δ*nrp* or *nrp*-comp strains were harvested, a cellular suspension was prepared, and the indicated immune cell populations were determined by flow cytometry. The gating strategy is shown in Fig. S2 in the supplemental material. (I) On day 20 postinfection, the expression of CCL2, CCL7, IFN-γ, and TNF was determined by real-time PCR, as described in Materials and Methods. Data are shown as the means ± SEMs from 5 independent animals in at least 2 independent experiments. The initial bacterial burdens were log_10_ 1.86 ± log_10_ 0.04460 and log_10_ 1.931 ± log_10_ 0.0822 for Δ*nrp* and *nrp*-comp strains, respectively. Statistical analysis was performed with a two-way ANOVA using Sidak’s test for multiple comparisons. *, *P* < 0.05; **, *P* < 0.01; ***, *P* < 0.001; ****, *P* < 0.0001.

So far, our data indicated that in the absence of *nrp*, mice had a better ability to control the infection at initial time points and showed a striking lesser pathology even at late time points of infection. These findings suggest that a differential immune response may be occurring in the absence of Nrp function. To test this hypothesis, we next investigated the dynamics of immune cell populations elicited during infection with Δ*nrp* or *nrp*-comp strains. We focused on myeloid (alveolar macrophages, inflammatory monocytes, and neutrophils) (Fig. 3A to C) and lymphoid (CD4^+^ T cells, CD8^+^ T cells, and B cells) (Fig. 3D to F) cells known to be relevant to the pathogenesis of TB. Whereas no substantial differences were found throughout the infection with either strain in alveolar macrophages (Fig. 3A), neutrophils (Fig. 3C), CD8^+^ T cells (Fig. 3E), and B cells (Fig. 3F), significantly fewer inflammatory monocytes (Fig. 3B) and CD4^+^ T cells (Fig. 3D) were detected at day 20 postinfection in the lungs of mice infected with the Δ*nrp* strain. These differences were attenuated by day 30 postinfection and were no longer present by day 90 postinfection (Fig. 3B and D). In addition to the decreased numbers of inflammatory monocytes and CD4^+^ T cells observed 20 days postinfection in the absence of *nrp* expression, the number of activated cells within these populations, as measured by the expression of surface markers major histocompatibility complex (MHC) class II and CD44, was also decreased (Fig. 3G and H). The gating strategy using in the definition of these populations is presented in Fig. S2.

10.1128/mSphere.00352-18.2 FIG S2 Gating strategy used in this study, for myeloid (A) and lymphoid (B) cell populations. Download FIG S2, TIF file, 0.7 MB. Copyright © 2018 Bhatt et al. 2018 Bhatt et al. This content is distributed under the terms of the Creative Commons Attribution 4.0 International license.

At the molecular level, the diminished recruitment of inflammatory monocytes seen in the Δ*nrp* strain may be explained by a lower expression of CCL2 and CCL7, chemokines that play a role in the recruitment of myeloid cells to the site of infection (21), in the lungs of these animals (Fig. 3I). In line with a lower frequency of activated CD4^+^ T cells in Δ*nrp*-strain-infected animals, a decreased expression of gamma interferon (IFN-γ) was observed in the lungs of these animals compared to that in *nrp*-comp-strain-infected mice (Fig. 3I). Tumor necrosis factor (TNF), another inflammatory mediator during TB, is also expressed at a lower level upon infection with the Δ*nrp* strain (Fig. 3I).

In all, the absence of *nrp* function in M. tuberculosis improves the host’s ability to control bacterial growth early postinfection, which is reflected in a lower bacterial burden. This is accompanied by diminished inflammatory monocyte and CD4^+^ T cell recruitment and activation, as well as decreased expression of inflammatory mediators, which in the long-term likely lower the progression of lung pathology. Thus, these data suggest Nrp as a novel virulence factor for M. tuberculosis, altering the initial phases of infection and the immune response, but with a long-lasting impact on the pathology developed at the site of infection.

### Δ*nrp* mutant is attenuated for growth in immune-deficient mice.

**FIG 4:**
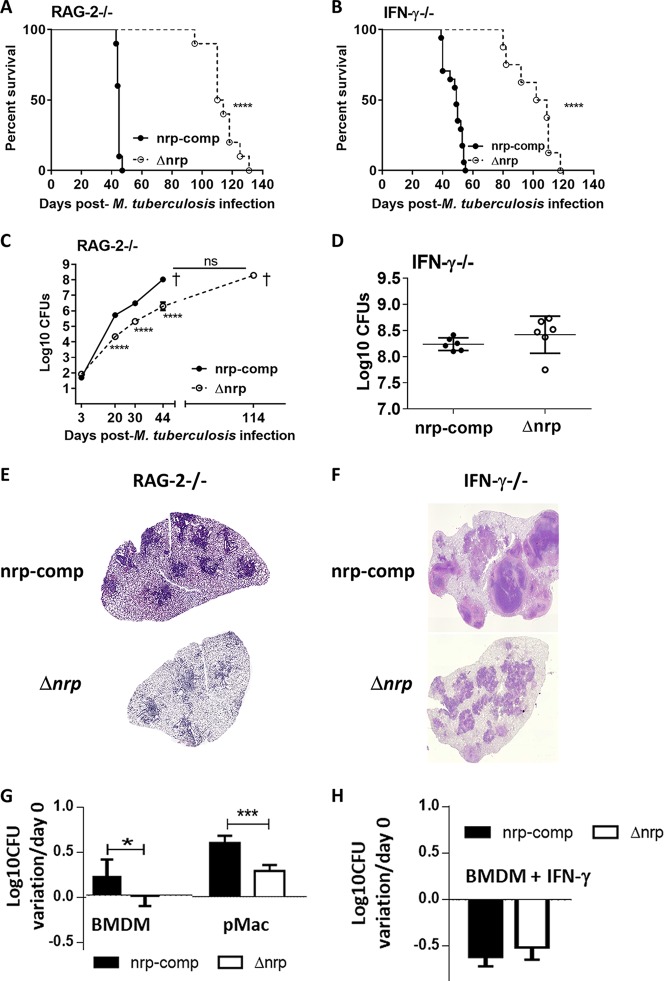
Growth of Δ*nrp* mutant is attenuated in immune-deficient mice. C57BL/6 RAG2^−/−^ (A, C, and E) and IFN-γ^−/−^ (B, D, and F) mice were infected by aerosol exposure to a low dose of the Δ*nrp* or *nrp*-comp strain. (A and B) The weights of the animals were monitored weekly up to day 30 and every 2 days thereafter to determine the survival curves. At the indicated time points (C) or at the time of death (D), the lungs of infected mice were collected and the bacterial burden was determined by CFU enumeration. The lung pathology was determined on day 40 postinfection for RAG2^−/−^ (E) or at the time of death for IFN-γ^−/−^ (F) animals. Shown are pictures from H&E staining for one animal representative of the experimental group; ×40 magnification. The initial bacterial burdens were log_10_ 1.92 ± log_10_ 0.069 and log_10_ 1.701 ± log_10_ 0.0707 (RAG2^−/−^ experiment) and log_10_ 1.92 ± log_10_ 0.069 and log_10_ 1.748 ± log_10_ 0.0707 (IFN-γ^−/−^ experiment) for Δ*nrp* and *nrp*-comp strains, respectively. BMDM (G and H) or pMac (G) were infected with Δ*nrp* or *nrp*-comp strains at an MOI of 1 in the absence (G) or presence (H) of exogenous IFN-γ. On day 4 postinfection, the intracellular bacterial load was determined by CFU enumeration. Data are shown as the means ± SEMs from 8 independent animals (A to F) or from 6 replicate wells in 2 independent experiments (G and H). Statistical analysis was performed with log-rank (Mantel-Cox) tests for the Kaplan-Meier curves (A and B) or two-way ANOVAs using Sidak’s tests for multiple comparisons (C to H). *, *P* < 0.05; ***, *P* < 0.001; ****, *P* < 0.0001.

Our findings point to a role for *nrp* during the early stages of M. tuberculosis infection. These findings led us to investigate for possible differences in the course of infection with Δ*nrp* or *nrp*-comp strains in immune-deficient mice. We first infected SCID mice with aerosols of WT H37Rv, Δ*nrp*, or *nrp*-comp strains and observed a remarkably longer survival rate for mice infected with the Δ*nrp* mutant (see Fig. S3). As with the above-described experiments performed with immunocompetent mice, given that the survival patterns were restored upon complementation, we performed all subsequent experiments with immunodeficient mice using the Δ*nrp* and Δ*nrp*-comp strains. Next, we infected recombination activating 2 (RAG2)-deficient (−/−) mice and IFN-γ^−/−^ mice. Both mouse strains are unable to control M. tuberculosis growth and typically succumb to infection 40 to 50 days after infection (22, 23). In line with this, both RAG2^−/−^ and IFN-γ^−/−^ mice succumbed to infection with the *nrp*-comp strain (Fig. 4A and B). Strikingly, both mouse strains infected with the Δ*nrp* mutant registered a significantly higher survival rate (average survival of 114 days against 44.7 days in RAG2^−/−^ mice and 99 days against 45 days in IFN-γ^−/−^ mice). An analysis of lung CFU counts indicated that the Δ*nrp* mutant grew at a lower rate in the lungs of infected RAG2^−/−^ mice (Fig. 4C). However, at the time of death, the lung bacterial burdens observed for each *nrp* strain were identical in both RAG2^−/−^ (Fig. 4C) and IFN-γ^−/−^ (Fig. 4D) animals. Furthermore, at the time of death, the lungs of RAG2^−/−^ (Fig. 4E) and IFN-γ^−/−^ (Fig. 4F) mice infected with the Δ*nrp* strain displayed significantly less pathology than their *nrp*-comp counterparts. Therefore, our data are indicative that in the absence of acquired immunity or IFN-γ, the attenuated M. tuberculosis growth due to the loss of *nrp* function was much more prolonged, associating again with reduced pathology. This set of data supports a more effective control of bacterial burden by the host’s innate immunity, in the presence of limited pathology, in the absence of *nrp* function.

10.1128/mSphere.00352-18.3 FIG S3 The growth of the Δ*nrp* mutant is attenuated in immune-deficient mice. SCID mice were infected by aerosol exposure to a low dose of H37Rv, Δ*nrp*, or *nrp*-comp strains. The weights of the animals were monitored to determine the survival curves associated with each infection. Statistical analysis of the Kaplan-Meier curves was performed with log-rank (Mantel-Cox) test. ***, *P* < 0.001. Download FIG S3, TIF file, 0.1 MB. Copyright © 2018 Bhatt et al. 2018 Bhatt et al. This content is distributed under the terms of the Creative Commons Attribution 4.0 International license.

### Primary macrophages are more efficient at controlling the growth of the Δ*nrp* strain.

Altogether, our findings suggest a more effective control of the Δ*nrp* strain by the host during the innate immune response. Since macrophages are one of the first types of cells to be infected by M. tuberculosis and to exert microbicidal functions (24), we questioned whether the expression of *nrp* impacted the bacterial control by infected macrophages. For this, we resorted to an *in vitro* system where mouse bone marrow-derived macrophages (BMDM) or macrophages isolated from the peritoneal cavity (pMac) were infected with either of the strains, and the intracellular bacterial burden was assessed 96 h later. This time point ensured macrophage viability, thus avoiding the phenotypes related to cell death, rather than intracellular bacterial growth. The growth of the *nrp*-comp strain at this time point, in either of the macrophage types, was higher than that observed for the Δ*nrp* strain (Fig. 4G). We then investigated the impact of exogenous IFN-γ on the ability of BMDM to control the *nrp*-deficient and -complemented M. tuberculosis strains. Exogenous IFN-γ improved the control of either of the strains by the macrophages, ablating the differences observed in bacterial growth (Fig. 4H). Of note, the macrophages infected with either of the M. tuberculosis strains showed similar patterns of cell viability and of cytokine production (see Fig. S4), suggesting a specific phenotype linked to bacterial control in the absence of Nrp. Altogether, these data support a virulence-associated role for Nrp in M. tuberculosis infection during the initial interaction with host macrophages. However, intriguingly, once the macrophages become fully activated, the host advantage over the Δ*nrp* M. tuberculosis strain was lost. These *in vitro* findings are in line with the *in vitro* data, where the attenuation of the *nrp*-deficient M. tuberculosis strain is particularly notable during the early phase of infection but lost once the acquired immune response is established.

10.1128/mSphere.00352-18.4 FIG S4 Infection of macrophages with Δ*nrp* strain does not alter the secretion of TNF, IL-1β, and IL-10 nor the cell viability. Macrophages were infected with either the *nrp*-comp or Δ*nrp* strain; at 24 h postinfection, cytokine production was detected in the culture supernatants by immunoassays. On days 2 or 4 postinfection, the cell viability was determined by trypan blue exclusion. Data are shown as the means ± SEMs from triplicate wells in one experiment, where macrophages were obtained from different mice. Statistical analysis was performed with *t* tests; *, *P* < 0.05. Download FIG S4, TIF file, 0.1 MB. Copyright © 2018 Bhatt et al. 2018 Bhatt et al. This content is distributed under the terms of the Creative Commons Attribution 4.0 International license.

## DISCUSSION

TB remains a devastating disease to mankind, with high human and economic tolls (25, 26). To efficiently tackle TB, an improvement of several current tools is needed, notably with regard to prevention, diagnosis, and treatment. Owing to the alarming drug resistance rates, which caused 480,000 cases of complicated TB in 2016 (1), there is a pressing need for the development of novel treatment strategies. In this context, a clearer understanding of the key virulence factors in M. tuberculosis may help in finding new vulnerable and druggable targets. Different experimental approaches have been pursued for the identification of essential M. tuberculosis genes (2, 4, 16), including target-based screening. In this study, we aimed at elucidating the role of the *nrp* gene in the pathogenesis of TB, resorting to a genetically engineered *nrp-*deficient mutant M. tuberculosis strain, as well as *in vitro* and *in vivo* models of infection.

Previous studies reporting that the *nrp* gene harbored by M. tuberculosis is essential for *in vivo* growth (16) and expressed during early time points postinfection (18) support a role for the cryptic metabolite produced by Nrp during infection. Furthermore, our *in silico* analysis showed that *nrp* is part of a cluster of genes that is rare among the mostly nonpathogenic rapid-growing NTMs and highly conserved among the slow-growing pathogenic species of the genus *Mycobacterium*. Overall, these findings support a role for the product of the *nrp* cluster in the pathogenesis of infection by *Mycobacterium* species.

A previous screen aimed at finding M. tuberculosis persistence mutants in isoniazid-treated mice identified a transposon mutant with an insertion upstream of *Rv0097*, a gene encoding a putative oxodoreductase and possibly also involved in the biosynthesis pathway of the cryptic metabolite associated with *nrp* (17). The infection of WT mice through an intravenous route showed that the transposon mutant was attenuated in lungs and spleen (17). This former study thus supports the notion that one or more genes in the *Rv0096-Rv0101* cluster play a role in survival during *in vivo* infection. In addition to the findings obtained with the transposon mutant (17), the study presented herein characterizes this operon in a more thorough way from *in silico* to biological data. Indeed, by using the aerosol route of infection, we show that the *nrp* cluster is associated not only with a better transient control but also with altered lung pathologies and demonstrate its ability to cause delayed mortality in infected immunocompetent mice. Accordingly, we show that in immunocompetent hosts, the *nrp*-deficient M. tuberculosis strain showed a delayed growth pattern in the lungs during the first 20 days of infection compared to that of its WT H37Rv and complemented counterparts. This delay in growth at the site of infection was accompanied by the delayed dissemination of the *nrp*-deficient M. tuberculosis strain to the spleens of the infected mice, improved survival rates, and a lessened pathology even at late stages of infection and in immunocompromised hosts. This is important, as lung pathology is a major cause of TB pathogenesis, needed for bacterial transmission and associated with posttherapy complications (27, 28). Thus, Nrp may be a bacterial modulator of lung pathology. It is possible that the decreased expression of inflammatory molecules, such as IFN-γ and TNF, in lungs infected with the *nrp*-deficient M. tuberculosis strain contributes to the observed decreased pathology.

Our findings collectively support a role for *nrp* during the early phases of the immune response and demonstrate that M. tuberculosis has evolved to express genes that manipulate innate defenses. This early role for Nrp during infection fits with a previous report showing that the *in vivo* expression of *nrp* is restricted to the early phases of infection (18). Accompanying the lower bacterial burden in the lungs of M. tuberculosis Δ*nrp*-infected mice, we found a delayed recruitment and activation of inflammatory monocytes and CD4^+^ T cells. However, the recruitment of other immune cell populations was similar between the two infections, possibly suggesting that the differences observed for inflammatory monocytes and CD4^+^ T cells did not result solely from the reduced bacterial burdens upon M. tuberculosis Δ*nrp* infection. The differential course of infection in terms of dissemination and pathology caused by the M. tuberculosis Δ*nrp* strain may be linked to the differential recruitment of inflammatory monocytes. Previous studies showed that the induction of CCL2 during mycobacterial infection is associated with the recruitment of permissive monocytes to the lesion, which then support bacterial growth (21, 29). It is interesting to speculate that, as in the absence of *nrp*, CCL2 induction is lower and fewer permissive monocytes are recruited, and so a lower bacterial burden and less dissemination are observed. On day 30 postinfection, these differences are lost, and similar numbers of inflammatory monocytes are present in the lungs of mice infected with either of the M. tuberculosis strains.

The most notable and interesting finding on the role of *nrp* in TB pathogenesis came from the observation that the lack of this gene highly attenuates M. tuberculosis infection in immunodeficient hosts. We were surprised to discover that RAG2^−/−^ , SCID^−/−^, and IFN-γ^−/−^ mice infected with the M. tuberculosis Δ*nrp* strain survived substantially longer than those infected with the complemented counterpart. As shown in the case of the RAG2^−/−^ mice, the growth of M. tuberculosis Δ*nrp* over time was much slower than that of the complemented strain. This suggests that in the absence of acquired immunity, the growth of M. tuberculosis Δ*nrp* remains in check, to the benefit of the host. A similar result is inferred from the infection of IFN-γ^−/−^ mice, as the bacterial burdens at the time of death were comparable between M. tuberculosis Δ*nrp* and its complemented counterpart. Most importantly, independent of the genetics of the mouse host, the lung pathology resulting from infection with M. tuberculosis Δ*nrp* was much less exuberant that that observed for the complemented strain. The bacterial burden phenotype of the M. tuberculosis Δ*nrp* strain is most likely mediated by the bacterial interaction with the macrophage. Indeed, we showed that primary mouse macrophages control the bacterial growth more efficiently when *nrp* is absent. However, the mechanistic bases of this better control remain unknown but do not seem to depend on the altered production of, at least, TNF, interleukin 1β (IL-1β), and interleukin 10 (IL-10). Further studies, perhaps encompassing global transcriptional analysis, are thus required to further explore this observation. As for the *in vivo* situation, once the macrophages became fully activated by IFN-γ, the impact of the absence of *nrp* is lost.

In all, our findings highlight a role for the *nrp* cluster in evading the basic microbicidal mechanisms of the macrophage, which is then overcome by the adaptive immune response. It is tempting to speculate that the mechanisms that enable this result from host-pathogen coevolution events. Several outstanding questions arise from this study: which is the mechanism hijacked by Nrp at the level of innate immunity? How does the acquired immune response abrogate the impact of Nrp in the macrophage? As an answer to these questions, the infection of mice deficient for Toll-like receptor 2 (TLR2), TLR4, IL-10, arachidonate 5-lipoxygenase (ALOX5), or arachidonate 15-lipoxygenase (ALOX15) with the M. tuberculosis Δ*nrp* strain showed the same phenotype as WT mice (see Fig. S5 in the supplemental material). Therefore, basic recognition mechanisms, as well as an imbalanced anti-inflammatory response, do not seem to be associated with the *nrp* phenotype. The precise metabolite produced by the *nrp* cluster in M. tuberculosis remains unidentified, as we were unable to detect an *nrp*-related metabolite in lab-grown cultures of M. tuberculosis, and future efforts will likely focus on the identification of the product *ex vivo* in infected tissue. Though challenging, these experiments will enlighten the biological role of the *nrp* cluster as well as the best timing to target it from a therapeutic point of view. Despite the importance of pursuing these questions in the future, our study provides strong evidence linking the *nrp* cluster to M. tuberculosis attenuation, specifically in the context of an immunocompromised host. Considering the huge impact of the HIV epidemics on TB, our data call for immediate efforts to be put toward a further understanding of the biological role of Nrp and the development of therapies targeted to immunocompromised TB patients.

10.1128/mSphere.00352-18.5 FIG S5 Competent TLR2, TLR4, IL-10, ALOX5, or ALOX15 expression is not required for the decreased bacterial burden seen during M. tuberculosis Δ*nrp* infection. TLR2-, TLR4-, IL-10-, ALOX5-, or ALOX15-deficient mice were infected with the Δ*nrp* strain in parallel with WT mice, and lung CFUs were measured on days 3 and 20 postinfection. As a control, CFUs were also measured in WT mice infected with the *nrp*-comp strain. Data represent the means ± SEMs from 3 (day 3) or 5 (day 20) independent mice. Download FIG S5, TIF file, 0.2 MB. Copyright © 2018 Bhatt et al. 2018 Bhatt et al. This content is distributed under the terms of the Creative Commons Attribution 4.0 International license.

## MATERIALS AND METHODS

### Comparative genomics.

Sequence identity scores were obtained by using the BLAST+ 2.6.0 (30) application to search a local database of 72 genomic sequences from species selected to be representative of the phylogeny of the genus *Mycobacterium* (19) (NZ_LQOM00000000, NZ_LQOQ00000000, NZ_LQOR00000000, NZ_LQOS00000000, NZ_LQOT00000000, NZ_LQOY00000000, NZ_ARBU00000000, NZ_LQPB00000000, NZ_LQPI00000000, NZ_ADNV00000000, NZ_LQPP00000000, NZ_CCAU000000000, NZ_MIHD00000000, NZ_LQQA00000000, CP000480, GCA_000243415.3_ASM24341v3, GCA_002102065.1_ASM210206v1, NC_000962, NC_002677, NC_002945, NC_008595, NC_008611, NC_008726, NC_010397, NC_010612, NC_014814, NC_015564, NC_015576, NC_015758, NC_015848, NC_016604, NC_016946, NC_018027, NC_018612, NC_019966, NC_020133, NC_021715, NC_022663, NZ_AGVE00000000, NZ_AJFI00000000, NZ_APKD00000000, NZ_CCBF000000000, NZ_CP007220, NZ_CP011269, NZ_CP011491, NZ_CP011530, NZ_CP014475, NZ_CP015278, NZ_CP020821, NZ_CSTD00000000, NZ_LQOJ00000000, NZ_LQOL00000000, NZ_LQOU00000000, NZ_LQOV00000000, NZ_LQOW00000000, NZ_LQOX00000000, NZ_LQOZ00000000, NZ_LQPC00000000, NZ_LQPD00000000, NZ_LQPE00000000, NZ_LQPF00000000, NZ_LQPG00000000, NZ_LQPH00000000, NZ_LQPJ00000000, NZ_LQPN00000000, NZ_LQPO00000000, NZ_LQPQ00000000, NZ_LQPR00000000, NZ_LQPS00000000, NZ_LQPT00000000, NZ_LQPU00000000, NZ_LQPW00000000, and NZ_LQPX00000000). The database was queried using the sequence of the *nrp* cluster (*Rv00097-Rv010*1) from H37Rv (NC_000962.3). The sequence identity score was calculated by combining the query coverage, E value, and identity values for each hit with the weights 0.5, 0.25, and 0.25, respectively. A multiple-genome alignment of the sequences NC_002677 (M. leprae), NC_015848 (Mycobacterium canettii), NC_002945 (Mycobacterium bovis) and NC_000962 (M. tuberculosis) was performed using Mauve 2.4.0 (31). The level of nucleotide diversity (π) was calculated with MEGA7.1.0 (32) using multiple-sequence alignments of 220 genomic sequences from MTBC strains (lineages 1 to 7) (20).

### Construction of a M. tuberculosis H37Rv *nrp* null mutant.

A null mutant of M. tuberculosis H37Rv *nrp* (*Rv0101*) was generated by Specialized Transduction using previously described protocols (33, 34). Briefly, a derivative of temperature sensitive phage phAE159 was constructed to replace *nrp* (phΔ*nrp*) with a hygromycin resistance gene (*hyg*). Hygromycin resistant colonies obtained following transduction of M. tuberculosis H37Rv were verified by Southern blot to confirm the chromosomal replacement of *nrp* with *hyg.* One such transductant was termed Δ*nrp* and used in all subsequent experiments.

### Generation of an *nrp-*complemented strain.

A 28-kb M. tuberculosis H37Rv cosmid fragment (spanning genomic position bp 104933 to bp 133536) was packaged in the integrative shuttle cosmid vector pYUB412-Kan to generate pYUB2420. This recombinant cosmid spans a region that extends from *Rv0096* to *Rv0109*. pYUB2420 was introduced into the Δ*nrp* strain by electroporation, and six kanamycin-resistant transformants were confirmed by Southern blot to contain chromosomally integrated pYUB2420. One of these transformants, termed *nrp*-comp, was selected for further studies.

### Bacterial growth and stocks.

Bacteria were grown in Middlebrook 7H9 liquid medium (BD Biosciences) supplemented with 0.05% Tween 80, 0.2% glycerol, and 10% oleic acid-albumin-dextrose-catalase (OADC) for 7 to 10 days and then subcultured in Proskauer Beck (PB) medium supplemented with 0.05% Tween 80 and 2% glycerol to the mid-log phase. Bacterial stocks were aliquoted and frozen at −80°C. To determine the concentration of M. tuberculosis in aliquots, 6 frozen aliquots were serial diluted and plated on Middlebrook 7H11 (BD Biosciences) agar plates supplemented with 10% OADC and 0.5% glycerol. Viable bacteria were determined by CFU enumeration after 21 to 28 days of incubation at 37°C.

### Ethics statement.

All animal experiments were performed in strict accordance with the recommendations of the European Union Directive 2010/63/EU. All experimental procedures were approved by the local animal ethics committee, and licensed by the Portuguese National Authority for Animal Health (DGAV, Portugal) with reference number 014811/2016-07-13. Animal procedures were conducted by laboratory personnel with accreditation for animal research given by DGAV. The mice were euthanized by CO_2_ inhalation, with efforts to minimize suffering.

### Mouse strains.

C57BL/6 mice were purchased from Charles River Laboratory (Barcelona, Spain). RAG2-deficient mice were purchased from Instituto Gulbenkian de Ciência (IGC) or generously provided by Margarida Correia-Neves (ICVS), and IFN-γ deficient mice were generously provided Susana Roque (ICVS). All transgenic mice were of a C57BL/6 genetic background. SCID mice were obtained from Charles River Laboratory (USA).

### Experimental infection.

Mice were infected with M. tuberculosis H37Rv (WT), Δ*nrp*, or *nrp*-comp strains via the aerosol route by using an inhalation exposure system (Glas-Col), according to previously published protocols (35, 36), which resulted in the delivery of 100 to 200 viable bacteria to the lungs.

### Survival.

The weights of the infected mice were determined every week after infection for immunocompetent mice and every week after infection for the first 30 days and every 48 h from day 30 postinfection onwards for immunodeficient hosts. Mice were humanely euthanized if they lost 20% of their maximum weight or upon losing responsiveness to physical stimulation. Whenever possible, the lungs of moribund animals were harvested for histology and bacterial burden assessment.

### Organ processing.

At the indicated time points postinfection, the mice were euthanized by CO_2_ asphyxiation, and the organs were aseptically excised and processed as described previously (35, 36). Briefly, the lungs were perfused by applying 10 ml phosphate-buffered saline (PBS) through the right ventricle of the heart to flush blood cells, and single-cell suspensions were prepared in complete Dulbecco’s modified Eagle medium (cDMEM; DMEM supplemented with 10% fetal bovine serum [FBS], glutamine, HEPES, and sodium pyruvate, all from Gibco). Single-cell suspensions were used for bacterial burden determination, flow cytometry analysis, and RNA extraction. The number of cells was counted using a Countess automated cell counter (Life Technologies).

### Bacterial burden determination.

The assessment of initial bacterial burden was performed 3 days postinfection by growing viable bacteria from whole-lung homogenates. For the other time points, lung and spleen single-cell suspensions were incubated with 0.1% saponin (Sigma-Aldrich) for 10 min to release intracellular bacteria. The CFU was determined by plating 10-fold serial dilutions of saponin-treated cell suspensions on Middlebrook 7H11 agar plates supplemented as described in “Bacterial growth and stocks.” BBL MGIT PANTA antibiotic mixture (BD Bioscience) was added to prevent the contamination of lung samples. Viable M. tuberculosis colonies were counted after 21 to 28 days of incubation at 37°C.

### Flow cytometry.

Lung cells (1 × 10^6^ to 2 × 10^6^) were stained for surface antigens for 30 min at 4°C. Stained cells were washed and then fixed overnight in PBS containing 2% paraformaldehyde. The following antibodies were used: CD3-PE (clone 145-2C11; eBioscience), CD4-PB (clone RM4-5; eBioscience), CD8-FITC (clone 5H10-1; BioLegend), CD44-PerCPCy5.5 (clone 1M7; eBioscience), CD62L-PECy7 (clone MEL-14; BioLegend), CD19-APC (clone eBio1D3; eBioscience), Ly6G-APC (clone 1A8; BioLegend), Ly6C-PerCPCy5.5 (clone AL-21; BD Pharmingen), MHC II-FITC (clone AMS-32.1; BD Pharmingen), CD11b-PE (clone M1/70; BioLegend), and CD11c-PB (clone N418; BioLegend). Samples were run on an LSRII flow cytometer with Diva software, and the data were analyzed using FlowJo version 10.1.r7 software. The total number of cells in each gate was calculated using the total number of cells determined by the Countess automated cell counter. The gating strategy used is shown in Fig. S2 in the supplemental material.

### RNA extraction, reverse transcription, and real-time PCR.

Total RNA from infected lungs was extracted with TRIzol reagent (Invitrogen), and cDNA was synthesized and analyzed by real-time PCR, as described previously (35, 36). Target IFN-γ and TNF mRNA expression was quantified using SYBR green (GrisP) and specific oligonucleotides and normalized to the ubiquitin mRNA levels. The expression of CCL2 and CCL7 was quantified using specific primer-probe sets (Applied Biosystems) and normalized to the expression of hypoxanthine phosphoribosyltransferase (HPRT).

### Histology analysis.

The right upper lobes of the lungs were fixed in PBS containing 3.7% formaldehyde, embedded in paraffin, sectioned in 2- to 3 -μm-thick slices, and stained with hematoxylin-eosin (35). The lung surface area of inflammation was measured using ImageJ software (version 1.50e; NIH). The percentage of total lung area involved with inflammation was calculated by dividing the cumulative area of inflammation by the total lung surface area for each sample.

### Infection of bone marrow-derived macrophages and peritoneal macrophages.

BMDM were differentiated from bone marrow cells obtained from the femurs and tibiae of C57BL/6 mice and cultured in the presence of L-cell conditioned medium, as described previously (37). pMac were obtained by intraperitoneally injecting 1 ml of thioglycolate into WT C57BL/6 mice. After 4 days, the mice were euthanized and the peritoneums were washed with PBS. In both cases, 1 × 10^6^ cells were infected with Δ*nrp* or *nrp*-comp strains at a multiplicity of infection (MOI) of 1 bacteria to 1 macrophage. Four hours after infection, the cells were washed 4 times with PBS (Gibco) to remove extracellular bacteria. The adherent cells were cultured at 37°C in 1 ml of cDMEM in the presence or absence of 100 U/ml of IFN-γ for 96 h. Four or ninety-six hours postinfection, 0.1% saponin (Sigma-Aldrich) in PBS was added to the wells, and the cells were incubated at room temperature (RT) for 10 min to release intracellular bacteria. The number of viable bacteria was determined by plating 10-fold serial dilutions of the saponin-treated cell suspensions on supplemented Middlebrook 7H1 agar, as described in “Bacterial growth and stocks.”

### Cytokine determination by ELISA.

TNF, IL-1β, and IL-10 concentrations in the supernatants of infected macrophages were determined 24 h postinfection by enzyme-linked immunosorbent assays (ELISAs) with commercially available kits (eBioscience), according to the manufacturer’s instructions. Cytokine levels from uninfected cells were below the assay level of detection (not shown).

### Cell viability assay.

Cell viability was determined in noninfected or infected macrophages 2 or 4 days postinfection. Macrophages were gently detached from low-adherence tissue culture plates using a cell scraper. Cell viability was assessed by using trypan blue exclusion.

### Statistical analysis.

Data were analyzed using GraphPad Prism 6. The differences between groups were analyzed with two-way analyses of variance (ANOVAs) using Sidak’s tests for multiple comparisons. Kaplan-Meier survival curves were analyzed with the log-rank (Mantel-Cox) test. Differences were considered significant at *P* values of ≤0.05.
