# Supplementary figures and images for: A Nonribosomal Peptide Synthase Gene Driving Virulence in Mycobacterium tuberculosis
Source: mSphere. 2018 Oct 31;3(5):e00352-18. doi: 10.1128/mSphere.00352-18 (PMC6211224; doi:10.1128/mSphere.00352-18)

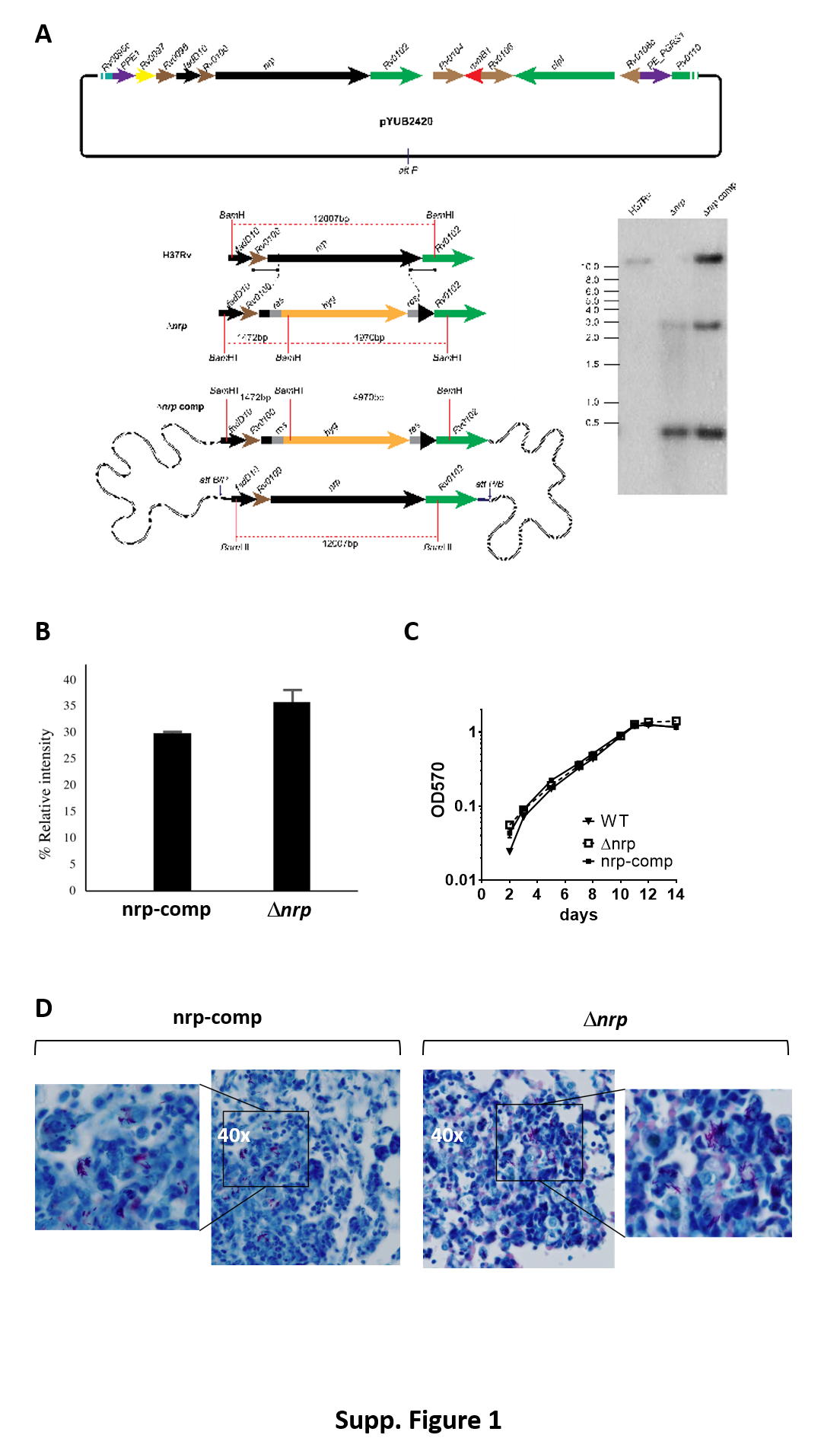

Supplement: FIG S1 [file sph006182687sf1.tif]

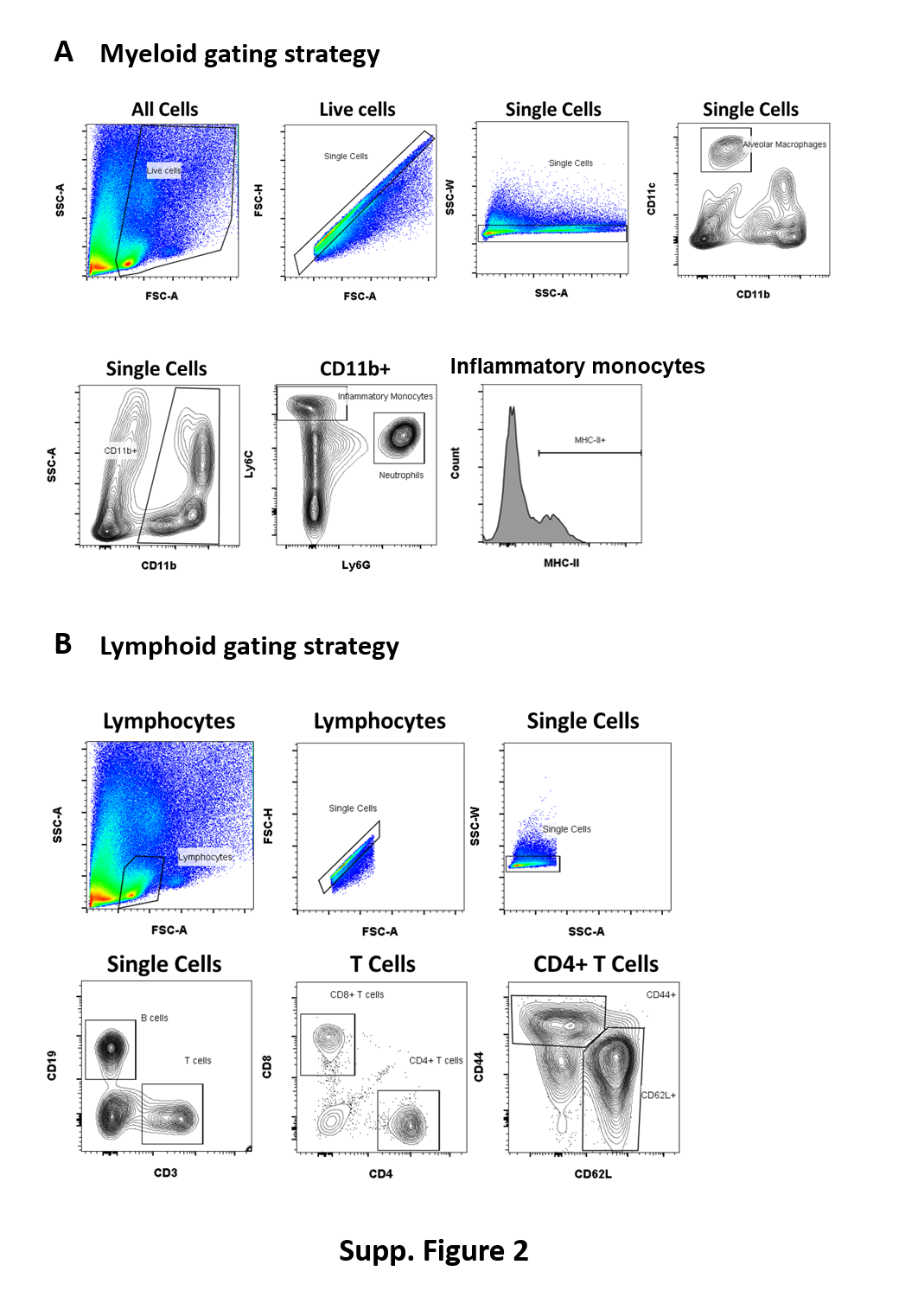

Supplement: FIG S2 [file sph006182687sf2.tif]

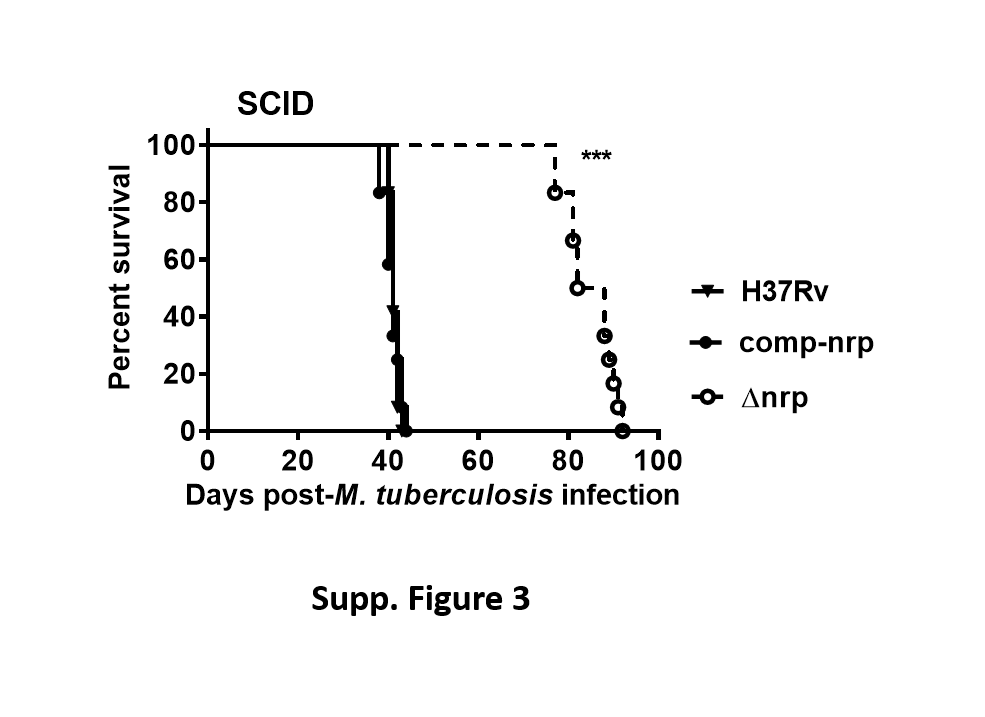

Supplement: FIG S3 [file sph006182687sf3.tif]

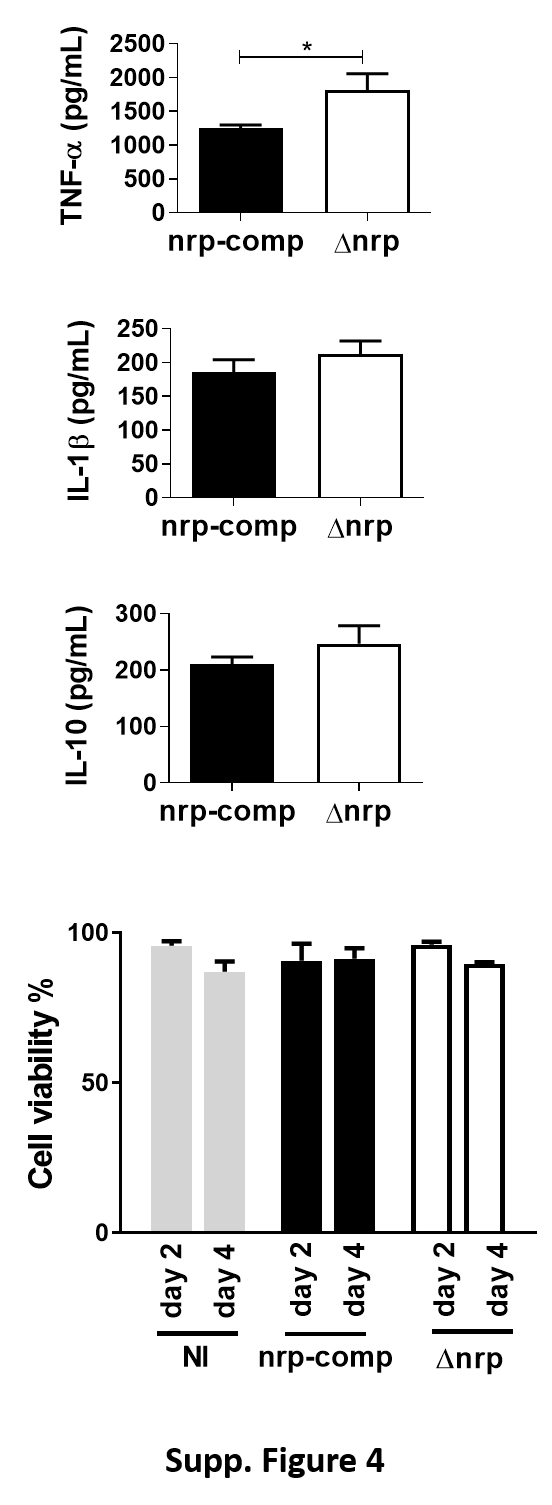

Supplement: FIG S4 [file sph006182687sf4.tif]

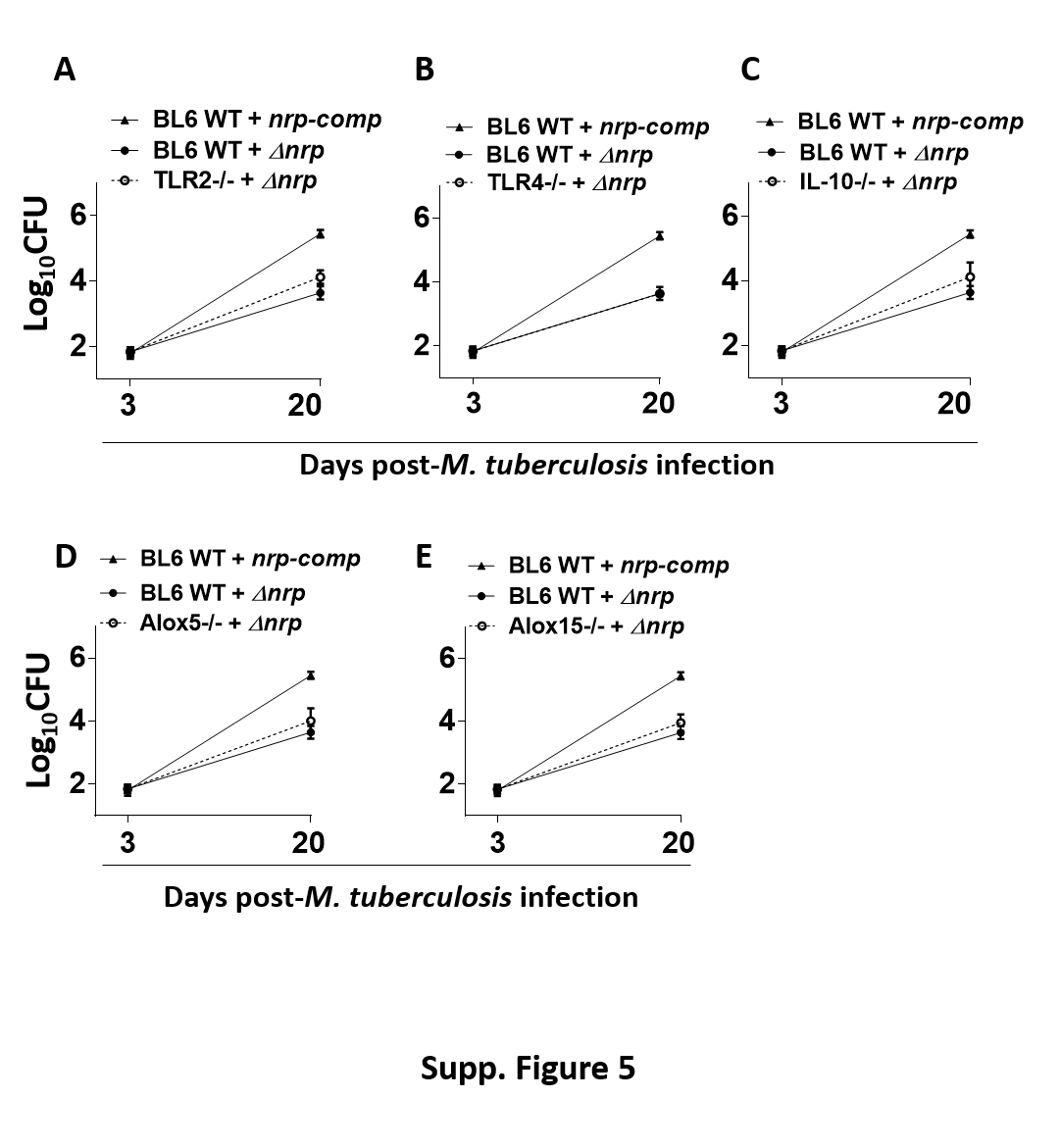

Supplement: FIG S5 [file sph006182687sf5.tif]
